# Auricular Composite Graft for the Reconstruction of a Partial Alar Defect: A Case Report

**DOI:** 10.7759/cureus.106574

**Published:** 2026-04-07

**Authors:** Yuto Yamamura, Kazuyasu Fujii, Kazutoshi Nishimura, Chisa Nakashima, Atsushi Otsuka

**Affiliations:** 1 Dermatology, Kindai University Hospital, Osaka, JPN

**Keywords:** auricular composite graft, basal cell carcinoma, cartilage graft, nasal ala reconstruction, partial alar defect

## Abstract

Reconstruction of partial nasal alar defects requires restoration of both aesthetic contour and functional support. For defects with preserved internal lining, local flaps and full-thickness skin grafts are commonly selected; however, local flaps may be challenging depending on the size and location of the defect, and skin grafts may be insufficient when the underlying structural support is compromised. We report a case of an octogenarian man with basal cell carcinoma of the nasal ala who underwent surgical excision followed by reconstruction using an auricular composite graft. The post-excisional defect measured approximately 1.6 cm in diameter and extended to a depth exposing the roots of the nasal hairs. A composite graft consisting of skin and cartilage was harvested from the posterior aspect of the auricle and transplanted to the defect. Although partial epidermal necrosis was observed postoperatively, complete epithelialization was achieved within approximately three weeks, with good graft survival and satisfactory aesthetic and functional outcomes. The donor site also healed without complications. Although auricular composite grafts are typically used for full-thickness alar defects, this case suggests that they may also be a useful option in selected partial defects requiring both skin coverage and structural support. This technique may represent a valuable single-stage reconstructive option that balances functional preservation, aesthetic outcome, and surgical invasiveness.

## Introduction

The nasal ala is a three-dimensional structure composed of skin, muscle, and fibro-fatty tissue, and its morphology is maintained by the support of these soft tissue components [1]. In addition, the nasal ala plays an important role in maintaining the patency of the nasal airway, and morphological alterations may adversely affect nasal function [2]. Thus, the nasal ala represents a structure of both functional and aesthetic importance, and its reconstruction requires restoration of both form and function.

In particular, when not only the skin but also the underlying supporting tissue is lost, there is a risk of depression and distortion of the alar contour [3]. An auricular composite graft, consisting of skin and cartilage harvested from the auricle, enables simultaneous restoration of cutaneous coverage and structural support. Auricular composite grafts have been primarily used for full-thickness alar defects and alar rim defects, where both skin and structural support are required [4].

On the other hand, for partial alar defects with preserved internal lining, local flaps or skin grafting are commonly selected [5]. Although auricular composite grafts have also been reported to be useful in such settings, their application in partial alar defects with preserved internal lining remains limited.

Herein, we report a case of a partial alar defect reconstructed using an auricular composite graft, which resulted in favorable aesthetic and functional outcomes.

## Case presentation

**Figure 1:**
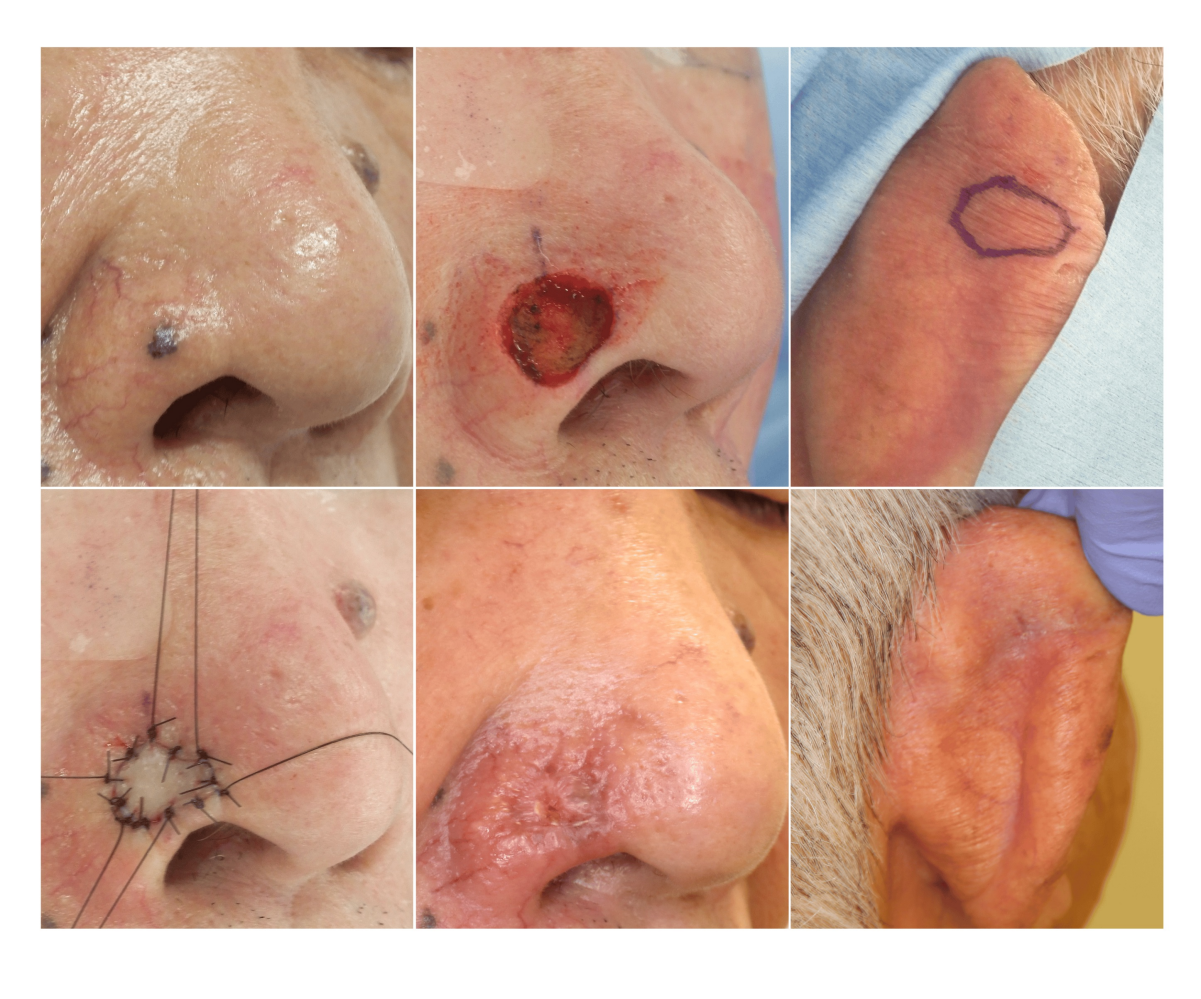
Clinical, intraoperative, and postoperative findings. (A) Preoperative photograph showing a black nodular lesion on the right nasal ala, including the surrounding nodules; the lesion measured approximately 1 cm in diameter. (B) Post-excisional defect measuring approximately 1.6 cm in diameter, with sufficient depth to require structural support reconstruction. (C) Harvesting of a composite graft consisting of skin and cartilage from the posterior aspect of the right auricle, selected to provide both cutaneous coverage and structural support. (D) The graft was sutured to the defect and secured with a tie-over dressing. (E) Postoperative appearance at two months. Although mild scar contracture was observed, graft survival was satisfactory, with favorable aesthetic and functional outcomes. (F) Donor site of the auricle at one month postoperatively, showing good healing.

An octogenarian man presented with a black nodular lesion on the nasal ala, which he had first noticed five to six years earlier. The lesion had gradually enlarged and had recently begun to bleed, prompting referral to our department. A partial biopsy revealed basal cell carcinoma, and surgical excision was planned (Fig. 1A).

The procedure was performed under general anesthesia. The tumor was excised with a 2-mm clinical margin (Fig. 1B). The resulting defect measured approximately 1.6 cm in diameter and extended to a depth exposing the roots of the nasal hairs. A composite graft consisting of skin and cartilage was harvested from the posterior aspect of the right auricle (Fig. 1C). The graft was then sutured into the defect and secured with a tie-over dressing (Fig. 1D). Histopathological examination confirmed nodular basal cell carcinoma with negative surgical margins.

Postoperatively, partial epidermal necrosis was observed; however, complete epithelialization was achieved within approximately three weeks. The graft survived well, with only mild scar contracture (Fig. 1E). The donor site healed uneventfully, and no functional impairment, such as nasal airway narrowing, was observed (Fig. 1F).

## Discussion

In the present case, an auricular composite graft was selected as the reconstructive method for a partial alar defect.

For reconstruction of partial alar defects, full-thickness skin grafting or local flap reconstruction is generally considered the standard approach [5]. However, in this case, not only the skin but also the underlying supporting tissue was lost. Therefore, reconstruction with a skin graft alone would not restore structural support and might result in depression or distortion of the alar contour due to scar contracture [2].

Furthermore, the nasal ala constitutes a critical component of the external nasal valve, and morphological alterations may lead to functional impairment, such as narrowing of the nasal airway [1]. In this case, considering the size and location of the defect, even if a local flap had been selected, reconstruction confined within the nasal subunit would likely have been difficult, potentially necessitating more invasive procedures such as a forehead flap [6].

Based on these considerations, an auricular composite graft was chosen in order to achieve an optimal balance between preservation of postoperative function and aesthetics and minimization of surgical invasiveness, as it enables simultaneous restoration of both skin coverage and structural support.

Auricular composite grafts are widely recognized as a reconstructive option for full-thickness alar defects and alar rim defects [4]. By contrast, for partial alar defects with preserved internal lining, local flaps or full-thickness skin grafts are more commonly selected.

This tendency may reflect the fact that skin grafting and local flap reconstruction are well-established and routinely performed techniques in reconstruction following excision of cutaneous tumors. In addition, composite grafts containing cartilage may be less frequently considered by clinicians due to concerns regarding graft survival and limited clinical experience with this technique.

Auricular composite grafts are relatively easy to harvest, and favorable healing of the donor site has been well documented [4]. Furthermore, because the auricle has a complex three-dimensional structure, it has been suggested that various defect configurations can be accommodated by adjusting the donor site and graft shape [7]. These characteristics make auricular composite grafts a potentially useful option for reconstruction requiring sufficient thickness or involving anatomically complex structures such as the nasal ala.

However, there are certain limitations. A graft size of approximately 1 cm or less is generally considered the safe upper limit, as the risk of necrosis increases with larger grafts [8]. Some reports suggest that the viable size of the graft may be extended under conditions of adequate vascularity at the recipient site. In the present case, satisfactory graft survival was achieved with a graft measuring approximately 1.6 cm.

Nevertheless, there is still no consensus regarding the upper limit of graft size, and further investigation through the accumulation of additional cases is warranted.

Furthermore, the ability to tailor the donor site and graft configuration allows auricular composite grafts to accommodate a wide range of defect morphologies. This versatility may support an expansion of its indications, particularly in the reconstruction of anatomically complex, three-dimensional structures such as the nasal ala.

## Conclusions

This case suggests that, in the reconstruction of partial alar defects, an auricular composite graft can serve as a valuable option when balancing preservation of postoperative function and aesthetics with surgical invasiveness. In selected cases where local flaps or full-thickness skin grafts are insufficient to restore structural support or where more invasive procedures should be avoided, this technique enables simultaneous restoration of both skin coverage and support in a single-stage procedure and may represent a practical alternative.
